# Evaluating segmental liver function using T1 mapping on Gd-EOB-DTPA-enhanced MRI with a 3.0 Tesla

**DOI:** 10.1186/s12880-017-0192-x

**Published:** 2017-03-01

**Authors:** Zhi-Peng Zhou, Li-Ling Long, Wei-Jia Qiu, Ge Cheng, Li-Juan Huang, Teng-Fei Yang, Zhong-Kui Huang

**Affiliations:** 1 Department of Radiology, The First Affiliated Hospital of Guangxi Medical University, Nanning, Guangxi 530021 People’s Republic of China; 2 Department of Radiology, Affiliated Hospital of Guilin Medical University, Guilin, Guangxi 541001 People’s Republic of China

**Keywords:** Gd-EOB-DTPA, T1 mapping, Liver function, MRI

## Abstract

**Background:**

Assessing the liver function provides valuable information to evaluate surgical risk and plan accordingly. Current studies focus on whole liver function evaluation. However, assessment of segmental liver function is equally important in the clinical practice. The purpose of this study was to investigate whether Gd-EOB-DTPA-enhanced MRI can evaluate the liver function of each segment by using T1 mapping at 3 Tesla MRI.

**Methods:**

One hundred three patients were classified into one of 4 groups: a normal liver function (NLF) group (*n =* 38), a liver cirrhosis with Child-Pugh A (LCA) group (*n =* 33), a liver cirrhosis with Child-Pugh B (LCB) group (*n =* 21), and a liver cirrhosis with Child-Pugh C (LCC) group (*n =* 11). All patients underwent Gd-EOB-DTPA-enhanced MRI scans. T1 relaxation times were measured on the liver superimposing T1 mapping images. Reduction rate (△%) of T1 relaxation time of the liver parenchyma were calculated.

**Results:**

After 20 min of Gd-EOB-DTPA enhancement, the T1 relaxation time of all liver segments in the LCC group were different from those in all the other groups, and more liver segments from the LCB and LCA groups different from the NLF group (*p <* 0.05). For the LCB group, the areas under the receiver operating characteristic curves (AUCs) of different liver segments for hepatobiliary phase (HBP) were 0.654-0.904 on T1 relaxation time, and 0.709-0.905 on △%. For the LCC group, the AUCs of different liver segments for HBP were 0.842–0.997 on T1 relaxation time, and 0.887–0.990 on △%.

**Conclusions:**

For LCB patients, segmental liver function evaluation is possible using Gd-EOB-DTPA-enhanced MRI T1 mapping. For LCC patients, all liver segments can be used to evaluate liver function and both T1 relaxation time and the △% of T1 relaxation time have good diagnostic performance.

**Electronic supplementary material:**

The online version of this article (doi:10.1186/s12880-017-0192-x) contains supplementary material, which is available to authorized users.

## Background

Liver function assessment plays a significant role in clinical practice, especially for surgeons predicting future remnant liver function after partial hepatectomy. Assessing the function of each segment of liver provides valuable information to evaluate surgical risk and plan accordingly.

In recent years, using MRI to evaluate liver function became possible with the clinical application of liver specific MRI contrast agents. Gadolinium ethoxybenzyl diethylenetriamine penta-acetic acid (Gd-EOB-DTPA) has recently come into use to assess liver lesions and function [1–5]. Gd-EOB-DTPA can be injected as an intravenous bolus and provides enhanced MRI phase information as an MRI non-specific gadolinium contrast. After 20 min, approximately 50% of the administered dose is transported into the hepatocytes and gets eventually excreted into the bile in normal functioning human livers [6–8]. Gd-EOB-DTPA clearance depends on the integrity of the hepatocytes, and decreased liver function leads to a decreased liver uptake of Gd-EOB-DTPA. Previous studies have shown the feasibility of using Gd-EOB-DTPA enhanced MRI to predict liver function by measuring liver parenchymal or biliary tract enhancement on hepatobiliary-phase (HBP) MR imaging, or by calculation of the liver/spleen signal contrast ratio [9–12]. However, MR signal intensity is not an absolute value and there exists a non-linear relationship with the gadolinium concentration. Moreover, MRI signal intensity also varies at different time points due to MRI technical factors, such as radiofrenquency amplification, receiver coil and sequences designed by different MRI systems [13, 14]. On the other hand, the T1 relaxation time is an absolute value, and in theory, it is directly related to the concentration of Gd-EOB-DTPA in the body. It can be directly measured on MRI T1 mapping and used for comparison between different acquisition times.

The Child-Pugh classification has been one of the most used means to estimate total liver function in the clinic. It consists of five clinical features and is used to evaluate the prognosis of chronic liver disease and cirrhosis [15, 16]. A study by Katsube in 2011 was the first one evaluating T1 mapping on Gd-EOB-DTPA-enhanced MRI to assess liver function [17], its results showed that post-contrast T1 relaxation times were significantly extended in cases of abnormal liver function compared to the shorter T1 relaxation times in normal livers. The 18-min time-point was deemed best to evaluate liver function. Other researchers have also shown correlations between the T1 relaxation time at the liver parenchyma and the liver function [18–23].

Current studies focus on whole liver function evaluation. However, assessment of segmental liver function is equally important in the clinical practice. The purpose of this study was to investigate whether Gd-EOB-DTPA-enhanced MRI can evaluate the liver function of each segment by using T1 mapping at 3 Tesla MRI.

## Methods

### Patients

This study was designed as a prospective study that included 103 consecutively enrolled patients who underwent GD-EOB-DTPA-enhanced MRI examination, at The First Affiliated Hospital of Guangxi Medical University and Affiliated Hospital of Guilin Medical University, from October 2014 to December 2015. This study was approved by the ethics committees of the First Affiliated Hospital of Guangxi Medical University and the Affiliated Hospital of Guilin Medical University. All patients signed informed consents before the contrast agent was injected.

The inclusion criteria consisted of the following: (1) patients with available clinical examination and biochemical tests that can be classified as a Child-Pugh score. (2) Absence of a liver resection surgery, radiofrequency ablation, chemotherapy or liver embolization procedure. (3) Absence of biliary obstruction or diffuse liver diseases caused by biliary tract disease. (4) The size of lesions was smaller than the segment which lesion existed.

As a result, 103 patients (82 men and 21 women, with a mean age of 54.2 ± 13.2 years) were included in the study. All patients were classified into one of 4 groups: a normal liver function (NLF) group (*n =* 38), a liver cirrhosis with Child-Pugh A (LCA) group (*n =* 33), a liver cirrhosis with Child-Pugh B (LCB) group (*n =* 21), and a liver cirrhosis with Child-Pugh C (LCC) group (*n =* 11); The characteristics of the patients in each group are shown in Table 1. For the groups comprised of patients with focal liver lesions, the size of the lesions had no effect on the T1 relaxation time measurements.

**Table 1:** Patient Characteristics

Group	NLF (*n =* 38)	LCA (*n =* 33)	LCB (*n =* 21)	LCC (*n =* 11)
Age (years)	53.9 ± 15.1	49.0 ± 13.3	55.2 ± 9.0	52.5 ± 12.4
Total bilirubin (umol/l)	15.7 ± 6.9	16.1 ± 7.4	48.4 ± 27.3	86.6 ± 21.2
Serum albumin (g/l)	42.3 ± 5.1	40.6 ± 4.2	33.5 ± 5.2	29.0 ± 3.5
Prothrombin time (INR)	1.05 ± 0.23	1.03 ± 0.23	1.22 ± 0.34	1.48 ± 0.59

Values = mean ± stardard deviation

### MRI

All patients underwent unenhanced and enhanced MRI scans (10 mL Gd-EOB-DTPA at 0.25 mmol/mL, Germany Bayer Healthcare Co.) using a Siemens Verio 3.0 T MRI scanner with a 12-channel body phased-array coil. Images were obtained with HASTE, TSE T2WI axial free breathing with fat suppression, EPI DWI axial breath hold with fat suppression, T1WI VIBE axial fat suppression plain and enhanced scanning. The Gd-EOB-DTPA was administered as a bolus, which was injected at a rate of 2 mL/s through the cubital vein; this was followed by a 20 mL saline chaser, which was administered at the same rate. For all patients, T1WI VIBE with syngo MapIt included: repetition time (TR) 3.9 ms, echo time (TE) 1.4 ms, flip angle 5° and 15°,field of view (FOV) 273 × 380 mm, Matrix 161X320 mm, 3 mm section thickness, and parallel imaging technique (*P =* 2) with generalized auto-calibrating partially parallel acquisition (GAPPA), performed for T1 mapping on pre-enhanced, 5, 10 and 20 min delay phases after GD-EOB-DTPA administration.

### Imaging analysis

**Fig. 1:**
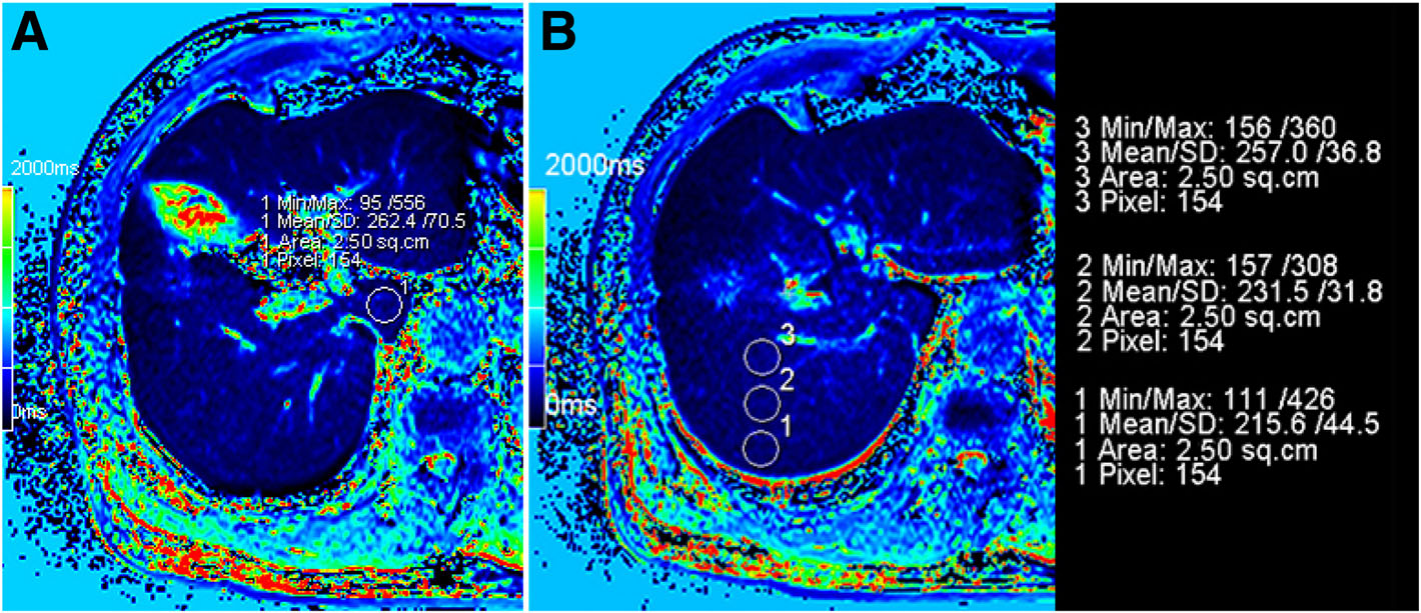
T1 relaxation measurement of liver segments, 1**a**: 1 ROI measurement in S1 segment;1**b**:3 ROI measurement in each liver segment

All the obtained data were transferred to a Siemens syngo workstation to measure T1 relaxation times using operator-defined regions of interest (ROIs). The ROI with a 2.15 cm^2^ (140–180 pixels) area was drawn manually on the liver superimposing T1 mapping images. Three ROIs were identified from the segment at the edge of the liver to the central liver segments S2-S8, one ROI was identified on liver segment S1, without focal lesions, major branches of portal or hepatic veins, or imaging artifacts. In addition, the rate of T1 relaxation time between pre-enhanced and post-enhanced phase at each time point was calculated using the following equation: Reduction rate (△%) = (T1_pre_-T1 _post_) ×100/T1_pre_, where T1_pre_ and T1_post_ were the T1 relaxation time of the liver segment before and after GD-EOB-DTPA administration (Fig. 1 and Additional file 1).

### Statistical analysis

The statistical analysis was performed using the SPSS 20.0 software package. Descriptive statistics (mean ± standard deviation) were provided when appropriate. The One-Way Anova least significant difference (LSD) analysis of variance was used to compare the differences in T1 relaxation time of liver segment for each group with the same segment. Receiver operating characteristic (ROC) curves were used to determine the diagnostic performance of T1 relaxation time and △% of T1 relaxation time for liver function. Corresponding areas under the ROC curve, sensitivities and specificities were calculated with 95% confidence interval (CI), the best cut-off value was predicted by the Maximum Youden-Index: Sensitivity + Specificity - 1. Any difference with a *p* value less than 0.05 was considered statistically significant.

## Results

### T1 relaxation time of each liver segment at different time points

The T1 relaxation times for each liver segment decreased gradually in the NLF and LCA groups from 5 min to 20 min after Gd-EOB-DTPA administration. The average T1 relaxation times at 20 min imaging delay ranged from 125.4 to 212.5 ms in the NLF group and from 138.4 to 228.2 ms in the LCA group. The T1 relaxation times for each liver segment varied unpredictably in the LCB and LCC groups from one time point to the next (5 to 20 min after the Gd-EOB-DTPA enhancement). The average T1 relaxation times at 20 min imaging delay ranged from 152.3 to 363.1 ms in the LCB group and from 315.6 to 485.4 ms in the LCC group (Additional file 2).

**Fig. 2:**
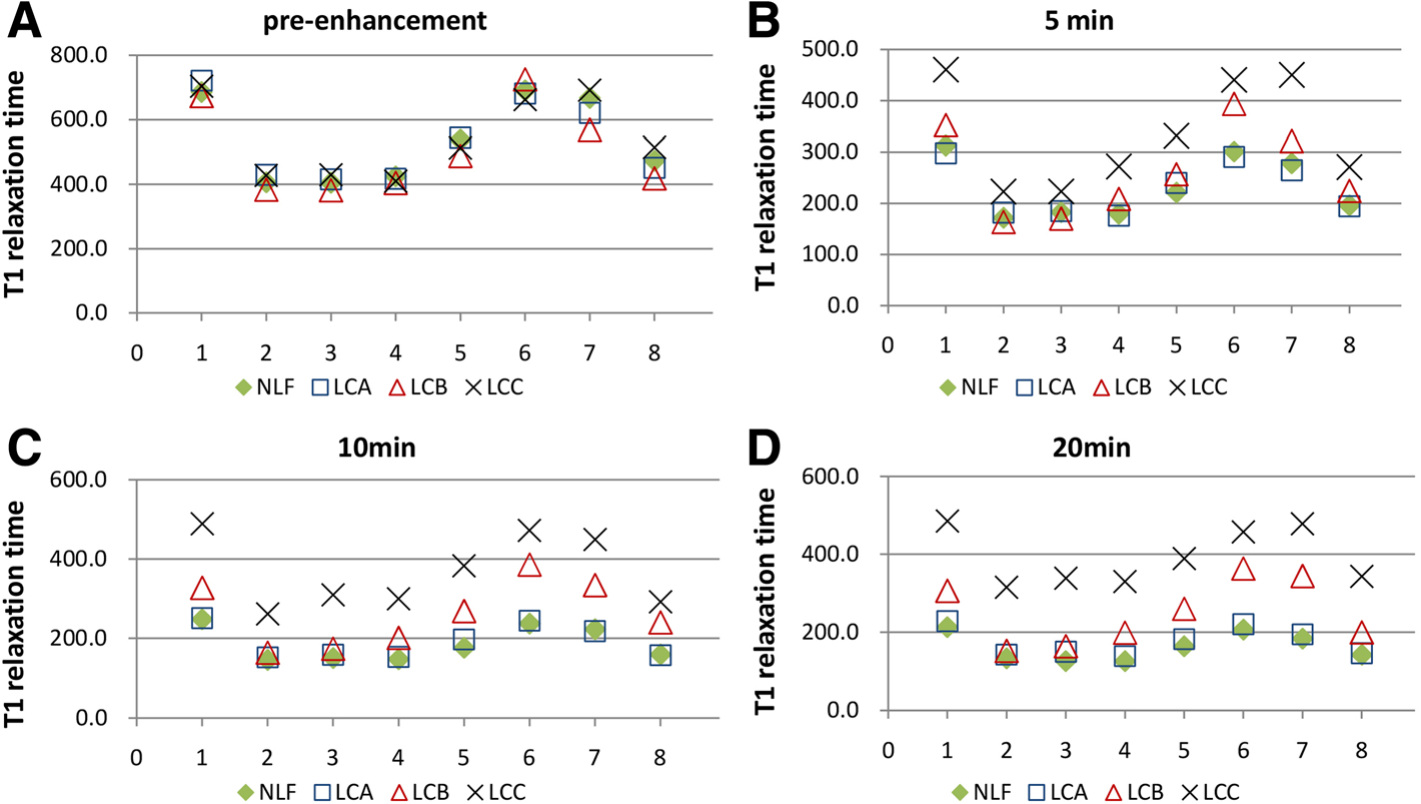
T1 relaxation time of each liver segment at different Gd-EOB-DTPA-enhanced MRI phases: 2**a**:T1 relaxation times in liver segments were no significantly different between groups before enhancement. 2**b**: After 5 min Gd-EOB-DTPA enhancement, the T1 relaxation times of the liver segments in the LCB and LCA groups were different from those in the NLF and LCA group (*p <* 0.05). 2**c**: After 10 min Gd-EOB-DTPA enhancement, T1 relaxation times of liver segments in LCB, and of all the segments in LCA were different from those in NLF and LCA group (*p <* 0.05). 2**d**: After 20 min Gd-EOB-DTPA enhancement, the T1 relaxation times in all LCA segments were different from the segments in the other groups, more liver segments in the LCB group were different from those in the NLF and LCA group (*p <* 0.05)

The T1 relaxation times in different segments of liver were not significantly different between groups before enhancement. After 5 min of Gd-EOB-DTPA enhancement, the T1 relaxation times in some segments started to differ, particularly most segments in the LCC group started to show markedly different T1 relaxation times than the liver segments of the other groups (*p <* 0.05). After 10 min of Gd-EOB-DTPA enhancement, more T1 relaxation times of liver segment became different among groups, some segments in the LCB group and all in the LCC group were significantly different from those in the NLF and LCA groups (*p <* 0.05). Finally, after 20 min of Gd-EOB-DTPA enhancement, the T1 relaxation time of all liver segments in the LCC group were different from those in all the other groups, and more liver segments from the LCB and LCA groups different from the NLF group (*p <* 0.05) (Fig. 2). The statistical results of T1 relaxation times of different liver segments among groups were provided in Table 2. The statistical results of the △% of T1 relaxation times of different liver segments among groups were provided in Table 3.

**Table 2:** One-Way Anova LSD test (significance level = 0.05) was used to compare the average T1rt on each liver segment for the four groups, the time points include pre-enhancement, 5, 10 and 20 min after GD-EOB-DTPA enhanced MRI

		S1	S2	S3	S4	S5	S6	S7	S8
Pre	p	0.575	0.382	0.533	0.915	0.492	0.684	0.103	0.284
	F	0.666	1.032	0.736	0.172	0.808	0.498	2.116	1.285
5 min	p	0.000	0.044	0.132	0.001	0.000	0.000	0.000	0.004
	F	9.010	2.789	1.917	6.323	7.869	10.287	16.486	4.748
10 min	p	0.000	0.000	0.000	0.000	0.000	0.000	0.000	0.000
	F	30.346	17.093	30.957	19.911	36.363	28.984	26.679	20.271
20 min	p	0.000	0.000	0.000	0.000	0.000	0.000	0.000	0.000
	F	30.960	34.918	63.449	38.156	38.245	38.883	35.003	42.828

Pre: Pre-enhancement; 5 min, 10 min and 20 min: the time point of post-enhancement, *p* value less than 0.05 was considered statistically significant; F: the ratio value of F test

**Table 3:** One-Way Anova LSD test (significance level = 0.05) was used to compare the average △% of T1rt on each liver segment for the four groups, the time points include 5, 10 and 20 min after GD-EOB-DTPA enhanced MRI

		S1	S2	S3	S4	S5	S6	S7	S8
5 min	P	0.000	0.148	0.397	0.001	0.000	0.000	0.000	0.001
	F	14.539	1.820	0.999	17.813	15.366	20.433	25.336	5.992
10 min	P	0.000	0.000	0.000	0.000	0.000	0.000	0.000	0.000
	F	30.070	18.218	30.019	32.019	57.027	24.752	40.254	17.071
20 min	P	0.000	0.000	0.000	0.000	0.000	0.000	0.000	0.000
	F	31.490	37.797	54.010	44.451	52.427	36.159	42.761	36.332

Pre: Pre-enhancement; 5 min, 10 min and 20 min: the time point of post-enhancement, p value less than 0.05 was considered statistically significant; F: the ratio value of F test

### Diagnostic performance of liver segment function assessment

The ROC curves of LCB and LCC groups were used to compare the diagnostic performance of T1 relaxation time and △% of T1 relaxation time for assessment of liver segment function. The corresponding sensitivity, specificity and best cut-off value were calculated. For the LCB group, different liver segments showed different diagnostic performances (Table 4 and Table 5). However, all liver segments consistently showed good diagnostic performance in the LCC group (Table 6 and Table 7).

**Table 4:** Diagnostic performance of T1rt at HBP for assessing LCB group segment function by receiver operating characteristic (ROC) analysis

	S1	S2	S3	S4	S5	S6	S7	S8
AUC	0.836	0.654	0.727	0.810	0.836	0.904	0.852	0.771
95% CI	0.75–0.92	0.52–0.78	0.63–0.83	0.71–0.91	0.75–0.92	0.84–0.96	0.77–0.93	0.67–0.87
Cut-offs:	238.0	143.7	132.1	148.5	228.1	233.1	215.9	130.1
Sensitivity	90.5%	71.4%	90.5%	81.0%	71.4%	100%	90.5%	100%
Specificity	71.2%	71.2%	61.6%	75.3%	83.6%	78.1%	75.3%	46.6%

**Table 5:** Diagnostic performance for △% of T1rt at HBP for evaluating LCB group segment function by receiver operating characteristic (ROC) analyses

	S1	S2	S3	S4	S5	S6	S7	S8
AUC	0.870	0.709	0.738	0.818	0.889	0.856	0.905	0.850
95% CI	0.99–0.94	0.58–0.84	0.63–0.85	0.72–0.92	0.81–0.96	0.78–0.93	0.84–0.97	0.76–0.94
Cut-offs:	63.9%	66.8%	70.7%	64.8%	47.9%	67.7%	37.1%	50.0%
Sensitivity	75.3%	63.0%	42.5%	71.2%	97.3%	63%	98.6%	94.5%
Specificity	100%	85.7%	95.2%	90.5%	66.7%	100%	71.4%	71.4%

**Table 6:** Diagnostic performance of T1rt at HBP for assessing LCC group segment function by receiver operating characteristic (ROC) analyses

	S1	S2	S3	S4	S5	S6	S7	S8
AUC	0.912	0.969	0.997	0.972	0.966	0.918	0.842	0.990
95% CI	0.79–1.00	0.93–1.00	0.99–1.00	0.93–1.00	0.93–1.00	0.86–0.98	0.89–0.99	0.97–1.00
Cut-offs:	376.7	250.8	239.4	276.1	306.9	322.3	306.9	236.1
Sensitivity	81.8%	90.9%	100%	90.9%	100%	100%	100%	100%
Specificity	95.7%	96.8%	96.8%	97.9%	93.6%	79.8%	86.2%	90.4%

**Table 7:** Diagnostic performance for △% of T1rt at HBP for evaluating LCC group segment function by receiver operating characteristic (ROC) analyses

	S1	S2	S3	S4	S5	S6	S7	S8
AUC	0.940	0.986	0.989	0.973	0.960	0.953	0.887	0.990
95% CI	0.89–0.99	0.97–1.00	0.91–1.00	0.94–1.00	0.92–0.99	0.91–0.99	0.82–0.95	0.97–1.00
Cut-offs:	55.5%	45.6%	37.6%	45.6%	40.6%	45.4%	51.3%	44.5%
Sensitivity	84%	91.5%	95.7%	88.3%	90.4%	88.3%	78.7%	92.6%
Specificity	100%	100%	100%	100%	100%	100%	100%	100%

## Discussion

For patients requiring a liver resection, the conventional evaluation method has included a clinical liver function exam combined with a liver volume assessment to evaluate the future remnant liver function after partial hepatectomy. Based on CT scan and liver volume measurements, researchers reported that the hepatocyte volume per unit of body weight was significantly correlated with indocyanine green (ICG) clearance test results and other parameters of normal liver function [24]. Others also reported that the ICG-parameters were in proportional relationship with hepatic parenchymal cell volume [25]. Those reports regarded the liver as a homogeneous organ. However, yet other studies showed pathological evidence for differences between different liver regions [26–28]. The lessons from all those preliminary studies made it clear that an assessment of segmental liver function is a necessary approach for most patients, and that estimation of whole liver function may not be accurate.

To the best of our knowledge, no study has yet reported the evaluation of liver segments function by using T1 relaxation time with Gd-EOB-DTPA-enhanced MRI. In our study, we measured T1 relaxation time and calculated the △% of T1 relaxation time for each liver segment. The best diagnostic value of T1 relaxation time at HBP was from 250.8 to 376.7 ms for the LCC group and from 130.1 to 238.0 ms for the LCB group. The best diagnostic value of △% of T1 relaxation time at HBP was from 40.6% to 55.5% for the LCC group and from 47.9 to 70.7% for the LCB group. The mechanism of the different T1 relaxation times in different liver segments is not clear. In our study, the △% of T1 relaxation time was calculated for reducing the impact of the differences of the pre-enhancement liver T1 relaxation times in different segments. However, differences in the △% of the T1 relaxation time were still found in each segment. Our results showed that the changing trends of the T1 relaxation time and the △% of T1 relaxation time were similar in all segments. However, the value of each segment was quite different. Different liver segments had different diagnostic values. A single value may not be good enough to evaluate both whole liver function and segmental liver function.

The diagnostic value of the T1 relaxation times found in research publications differ from one to another. Katsube et al. [17] reported that the cut-off value of T1 relaxation time at 18 min after Gd-EOB-DTPA injection, to distinguish LCB from other groups with best accuracy is less than 520 ms at HBP. On the other hand, Haimerl et al. [20] reported that the cut-off to differentiate LCB from LCA was 329.5 ms. Yet, Ding et al. [18] reported that the HBP T1 relaxation time was equal to the △% of T1 relaxation time, and the results showed the post-contrast value of T1 relaxation time to be 292.3 ± 59.2 ms for poor liver function and 217.3 ± 52.9 ms for good liver function. Those reports showed significant differences in terms of the diagnostic value of T1 relaxation time for evaluation of liver function.

In our study, the diagnostic performance of T1 relaxation time and the reduction of T1 relaxation time differ in the different groups. For the LCC group, the AUC of diagnosis performance was above 0.9 for both T1 relaxation time and the △% of T1 relaxation time, but excluding the S7 (AUC = 0.84) measurement for T1 relaxation time at HBP. All liver segments showed a significant good performance of diagnosis for HCC patients. For the LCB group, the AUC of diagnostic performance for S6 and S7 measurement of T1 relaxation time were over 0.85, others segments have lower diagnostic performance AUC from 0.654 to 0.836. The results showed that for LCB patients, the three best segments for determining the diagnosis were S5, S6 and S7. S1 showed a similar diagnostic performance to that of S5. However, the size of S1 is much smaller than others liver segments, thus it is not a good option for making diagnostic measurement. The △% of T1 relaxation time measurement at HBP showed improved AUCs in each segment, except in the AUC of S6 that was reduced from 0.904 to 0.856. Our results showed that the △% of T1 relaxation time improved the diagnostic performance at HBP. Furthermore, the diagnostic value of T1 relaxation time has shown significant variation among different previous reports, but the diagnostic value of the △% of T1 relaxation time has shown variation only within a small range. So, we suggest that the △% of T1 relaxation time may be more efficient for evaluation of liver function.

Our study has several limitations. First, we only used the Child-Pugh score to classify liver function and assign patients to each group; no pathological proofs or ICG tests were included, so the liver function in each patient could differ markedly. Second, all patients were injected with the same dose of Gd-EOB-DTPA, no correlation between the dose and body weight may be a source of variation. Third, the results showed the difference of T1 relaxation time and the △% of T1 relaxation time in each liver segment after Gd-EOB-DTPA administration, although a correlation of T1 relaxation time and liver function at HBP was reported previously, we did not find any direct evidence that the T1 relaxation time in the HBP matched the real segmental liver function. Further studies are required.

## Conclusions

For LCB patients, segmental liver function evaluation is possible by using Gd-EOB-DTPA-enhanced MRI T1 mapping, and calculation of the △% of T1 relaxation time may be more efficient for evaluation of segmental liver function. For LCC patients, all liver segments can be used to evaluate liver function and both T1 relaxation time and the △% of T1 relaxation time have good diagnostic performance.

## Additional files

Additional file 1: Measurement of T1 relaxation time in all groups. A-D: measurement of T1 relaxation time in NLF group (A-D), LCB group (E-H) and LCC group (I-L), all images were obtained from pre-enhancement (A,E,I) 5 min (B,F,J), 10 min (C,G,K) and 20 min (D,H,L) after Gd-EOB-DTPA administration. The averages of T1 relaxation time were as follows: 630.2 ms (A), 225.0 ms (B), 166.6 ms (C), 160.1 ms (D), 846.0 ms (E), 314.7 ms (F), 248.7 ms (G), 226.3 ms (H), 504.5 ms (I), 246.5 ms (J), 273.4 ms (K), 288.5 ms (L). The reduction of T1 relaxation times at 5 min, 10 min and 20 min post-enhancement were 64.3%, 73.6% and 74.6% in NLF, 51.3%, 61.5 and 65.0% in LCB, and 51.1%, 45.8% and 42.8% in LCC, respectively. (PDF 1198 kb)
Additional file 2: The value of the T1 relaxation time and the percentage of △% of T1 relaxation time cross the all segment on pre- and post-enhancement T1 mapping in all groups. Additional table 1 showed that the value of the T1 relaxation time (ms) (Mean ± SD) cross the all segment on pre- and post-enhancement T1 mapping in all groups. Additional table 2 showed that the percentage of % of T1 relaxation time (%) (Mean ± SD) cross the all segment on pre- and post-enhancement T1 mapping in all groups. (PDF 270 kb)

## Abbreviations

△%: Reduction rate (of T1 relaxation time)
AUC: Area under the ROC curve
Gd-EOB-DTPA: Gadoxetic acid
HBP: Hepatobiliary phase
ICG: Indocyanine green
LCA: Liver cirrhosis with Child-Pugh A
LCB: Liver cirrhosis with Child-Pugh B
LCC: Liver cirrhosis with Child-Pugh C
MRI: Magnetic resonance imaging
NLF: Normal liver function
ROC: Receiver operating characteristic
ROI: Region of interest
