# Supplementary material for: Evaluating segmental liver function using T1 mapping on Gd-EOB-DTPA-enhanced MRI with a 3.0 Tesla
Source: BMC Med Imaging. 2017 Mar 1;17:20. doi: 10.1186/s12880-017-0192-x (PMC5333450; doi:10.1186/s12880-017-0192-x)

The next figure was just for review, those data were not included in manuscript considering the restricted length of manuscript.

Additional-Fig 1. A-D: measurement of T1 relaxation time in NLF group (A-D), LCB group (E-H) and LCC group (I-L), all images were obtained from pre-enhancement (A,E,I) 5 min (B,F,J), 10 min (C,G,K) and 20 min (D,H,L) after Gd-EOB-DTPA administration. The averages of T1 relaxation time were as follows: 630.2 ms (A), 225.0 ms (B), 166.6 ms (C), 160.1 ms (D), 846.0 ms (E), 314.7 ms (F), 248.7 ms (G), 226.3 ms (H), 504.5 ms (I), 246.5 ms (J), 273.4 ms (K), 288.5 ms (L). The reduction of T1 relaxation times at 5min, 10min and 20min post-enhancement were 64.3%, 73.6% and 74.6% in NLF, 51.3%, 61.5 and 65.0% in LCB, and 51.1%, 45.8% and 42.8% in LCC, respectively.

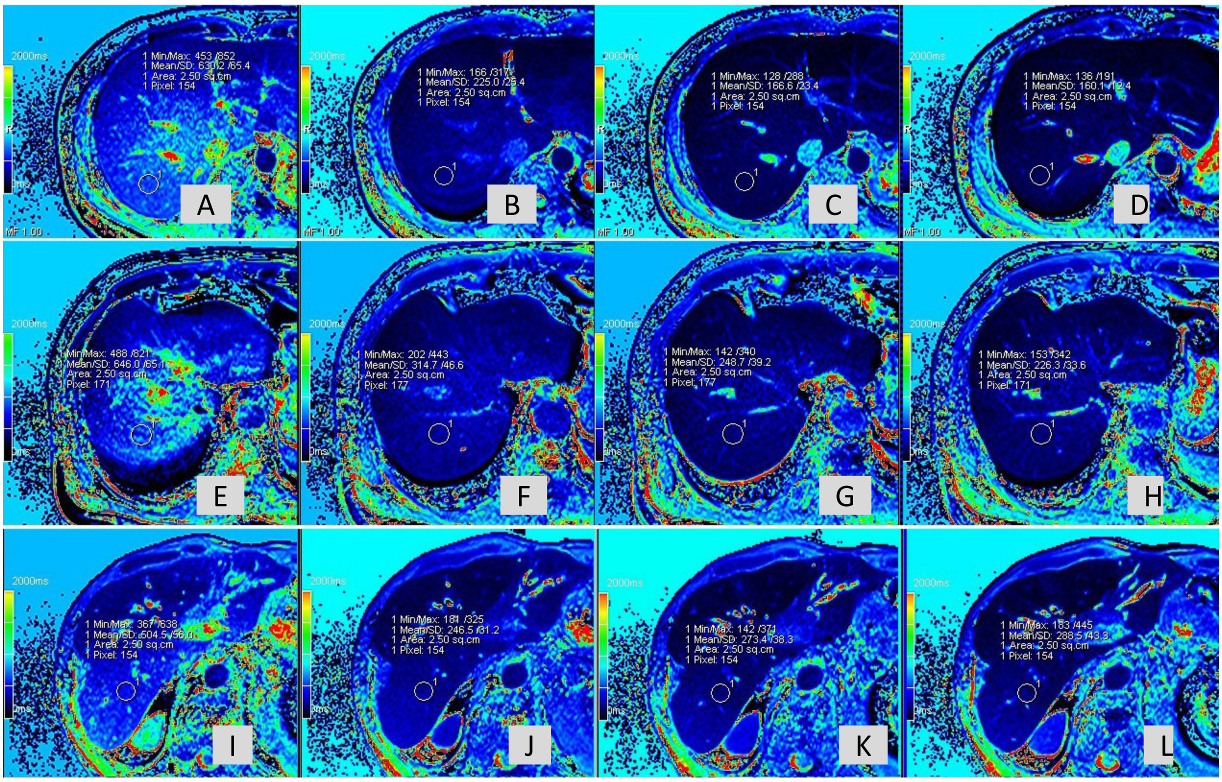

Supplement: Additional file 1: — Measurement of T1 relaxation time in all groups. A-D: measurement of T1 relaxation time in NLF group (A-D), LCB group (E-H) and LCC group (I-L), all images were obtained from pre-enhancement (A,E,I) 5 min (B,F,J), 10 min (C,G,K) and 20 min (D,H,L) after Gd-EOB-DTPA administration. The averages of T1 relaxation time were as follows: 630.2 ms (A), 225.0 ms (B), 166.6 ms (C), 160.1 ms (D), 846.0 ms (E), 314.7 ms (F), 248.7 ms (G), 226.3 ms (H), 504.5 ms (I), 246.5 ms (J), 273.4 ms (K), 288.5 ms (L). The reduction of T1 relaxation times at 5 min, 10 min and 20 min post-enhancement were 64.3%, 73.6% and 74.6% in NLF, 51.3%, 61.5 and 65.0% in LCB, and 51.1%, 45.8% and 42.8% in LCC, respectively. (PDF 1198 kb) [file 12880_2017_192_MOESM1_ESM.pdf]
