# Supplementary material for: Evaluating segmental liver function using T1 mapping on Gd-EOB-DTPA-enhanced MRI with a 3.0 Tesla
Source: BMC Med Imaging. 2017 Mar 1;17:20. doi: 10.1186/s12880-017-0192-x (PMC5333450; doi:10.1186/s12880-017-0192-x)
Supplement: Additional file 2: — The value of the T1 relaxation time and the percentage of △% of T1 relaxation time cross the all segment on pre- and post-enhancement T1 mapping in all groups. Additional table 1 showed that the value of the T1 relaxation time (ms) (Mean ± SD) cross the all segment on pre- and post-enhancement T1 mapping in all groups. Additional table 2 showed that the percentage of % of T1 relaxation time (%) (Mean ± SD) cross the all segment on pre- and post-enhancement T1 mapping in all groups. (PDF 270 kb) [file 12880_2017_192_MOESM2_ESM.pdf]

The next two tables was just for review, those data were not included in manuscript considering the restricted length of manuscript.

Additional-Table1. The value of T1 relaxation time (ms) (Mean±SD) cross the all segment on pre- and post-enhancement T1 mapping in all groups.

		S1	S2	S3	S4	S5	S6	S7	S8
NLF	pre	685±128	406±112	405±104	424±86	538±174	690±146	667±177	473±150
	5min	313±111	171±69	182±68	179±77	221±72	301±103	278±69	196±67
	10min	248±55	145±49	150±49	147±36	176±48	237±65	223±74	159±56
	20min	213±57	133±56	125±42	125±42	163±53	206±75	183±84	141±57
LCA	pre	721±122	430±107	415±100	418±113	545±133	681±151	621±169	451±173
	5min	297±70	182±57	184±58	175±58	240±72	291±86	264±67	194±73
	10min	251±86	152±49	159±55	153±76	197±69	244±94	218±73	157±59
	20min	228±87	142±53	150±55	138±66	182±70	221±90	195±72	146±59
LCB	pre	674±177	384±76	382±78	405±126	488±126	726±205	569±162	419±79
	5min	352±79	163±29	170±22	208±60	257±60	394±114	321±110	224±52
	10min	328±61	164±24	174±21	201±60	269±77	386±126	335±127	240±81
	20min	306±60	152±40	164±33	199±63	260±79	363±144	344±143	199±45
LCC	pre	703±123	428±86	430±108	410±107	512±17	662±125	691±86	515±69
	5min	461±130	223±71	223±90	272±90	331±68	441±95	450±91	271±56
	10min	489±146	261±76	310±72	300±72	382±59	472±92	449±88	292±58
	20min	485±184	315±73	339±57	330±82	389±77	458±59	478±121	343±56

Additional-Table 2. The percentage of △% of T1 relaxation time (%) (Mean±SD) cross the all segment on pre- and post-enhancement T1 mapping in all groups.

		S1	S2	S3	S4	S5	S6	S7	S8
NLF	5min	54±14	57±14	56±14	58±13	57±13	56±13	57±9	58±9
	10min	63±9	63±11	62±10	64±11	66±9	63±18	66±12	65±13
	20min	68±9	67±11	69±42	69±13	69±10	69±13	71±12	68±12
LCA	5min	59±7	58±9	55±10	58±8	56±10	57±7	56±10	55±14
	10min	65±11	64±11	61±12	64±12	64±10	64±10	64±11	62±14
	20min	68±11	66±12	63±13	67±12	66±10	68±10	67±15	66±11
LCB	5min	46±11	57±8	54±8	47±11	47±6	45±10	44±9	46±10
	10min	49±14	56±8	52±13	48±16	44±13	46±14	41±11	42±18
	20min	52±15	59±12	55±15	48±19	45±15	47±17	39±18	52±10
LCC	5min	35±14	49±11	49±18	35±10	35±8	34±9	35±8	48±8
	10min	31±16	39±10	27±8	27±14	25±12	29±7	35±8	44±4
	20min	32±21	26±11	20±10	19±11	24±16	30±10	31±12	33±6
